# Visit of Prof. Liebig

**Published:** 1850-09

**Authors:** 


*Visit of Prof. Liebig.*—It is stated in some of our exchange Journals, that Prof. Liebig is soon to visit the United States, and will probably give a series of lectures in various cities.

